# The Efficacy of a Ferric Sillen Core-Linked Polymer in Suppressing the Pathogenicity of *Campylobacter jejuni*

**DOI:** 10.3390/ani14213150

**Published:** 2024-11-02

**Authors:** Seán Christanseen, Dermot Walls, Blánaid White, Richard Murphy, Karina Horgan

**Affiliations:** 1 Alltech Ireland, Summerhill Road, A86 X006 Dunboyne, Ireland; rmurphy@alltech.com (R.M.);; 2 School of Biotechnology, Dublin City University, D09 V209 Dublin, Ireland; 3 National Centre for Sensor Research, Dublin City University, D09 E432 Dublin, Ireland; blanaid.white@dcu.ie; 4 DCU Water Institute, Dublin City University, D09 K20V Dublin, Ireland; 5 School of Chemical Sciences, Dublin City University, D09 E432 Dublin, Ireland

**Keywords:** *Campylobacter jejuni*, ferric sillen core-linked polymer, virulence factors, porcine jejunal epithelial (IPEC-J2), poultry pathogen

## Abstract

*Campylobacter* spp. are considered the leading bacterial cause of human gastroenteritis in the world. The development of effective intervention strategies aimed at limiting *C. jejuni* infections has encountered various challenges, including a lack of an appropriate animal model. Nevertheless, recent advancements in research have clarified the molecular mechanisms underlying *C. jejuni*’s pathogenicity, potentially opening new avenues for targeted interventions. This study evaluated the efficacy of a ferric sillen core-linked polymer (FSCLP) in lowering the proliferation and gene expression of *C. jejuni* virulence factors in vitro. Furthermore, this study sought to examine the impact of this FSCLP in an ex vivo environment by investigating its ability to influence the attachment to and invasion of porcine jejunal epithelial (IPEC-J2) cells by *C. jejuni*. Findings show that the FSCLP exhibits significant inhibitory effects on the growth of *C. jejuni* (*p* < 0.001) and decreases gene expression related to both virulence and colonisation in *C. jejuni.* Moreover, supplementation with the FSCLP significantly reduced the attachment of *C. jejuni* to IPEC-J2 cells (*p* < 0.01) when compared to the control. Thus, this water-soluble product presents a potential management strategy for *Campylobacter* infections in poultry, potentially impeding colonisation, reducing transmission, and ultimately mitigating the incidence of human campylobacteriosis.

## Simple Summary

This study evaluates the efficacy of a ferric sillen core-linked polymer (FSCLP) in controlling *Campylobacter jejuni* in-vitro, a leading cause of human gastroenteritis. The study focuses on the compounds ability to inhibit bacterial growth and reduce the expression of virulence-related genes. The FSCLP significantly decreases (*p* < 0.01) *C. jejuni* growth and attachment of the *C. jejuni* to intestinal porcine epithelial cells which is critical for colonisation. Moreover, gene expression analysis revealed downregulation of key genes involved in iron metabolism, adhesion, and toxin production after treatment with the FSCLP. These findings suggest that this FSCLP could serve as a potential antimicrobial strategy to manage *C. jejuni* infections, particularly in poultry, reducing bacterial transmission and, consequently, infection rates. Further research, particularly in vivo studies, are necessary to confirm its efficacy in real-world agricultural settings.

## 1. Introduction

Unlike other zoonotic diseases, the rate of *Campylobacter* infections worldwide has been increasing, with the number of cases often exceeding those of salmonellosis and shigellosis, resulting in campylobacteriosis being the most common cause of bacterial gastroenteritis in the developed world [1]. It is estimated globally that *Campylobacter* spp. cause approximately 96 million cases of food-borne illness each year [2]. About 90% of all Campylobacteriosis are caused by *Campylobacter jejuni* [3].

*C. jejuni* is a microaerophilic bacterium with specific physiological characteristics that distinguish it from other foodborne pathogens [4]. Its proliferation is limited to a narrow range of environmental conditions, requiring a microaerobic atmosphere, typically with oxygen levels of 3–5%, and thrives within a narrow temperature range of 37 °C to 42 °C [5]. This theoretical limitation suggests that *C. jejuni* cannot exist outside the host in a natural aerobic environment [6]. However, *C. jejuni* exhibits exceptional resilience, surviving in a variety of environmental reservoirs, including water supplies and food items, despite food items typically undergoing difficult processing conditions such as preservation, temperature changes, stress, and different pH levels [7].

*Campylobacter* virulence is a multifactorial process which includes the motility, chemotaxis, adhesion, invasion, translocation, production of toxins, and secretion of proteins [8]. The modulation of these virulence factors has been identified as a critical target in the development of alternative strategies for controlling these pathogenic microorganisms. One such alternative within the agricultural industry that has been used for decades is the use of bioactive compounds such as organic acids [9]. The antimicrobial mode of action of these compounds is based on their ability to cross the cell membrane, due to the lipophilic nature of their undissociated form, in the process of which the proton and associated anion concentrations in the cytoplasm of the bacterium are altered [10]. However, given that the ceca are considered the main reservoir for *Campylobacter* and, due to most organic acids dissociating prior to reaching the lower gastro-intestinal tract (GIT), organic acids alone may not be the answer to this issue [11]. One way to resolve this issue is to increase organic acid stability through the exploitation of its chelating ability with transition metals. Recent studies [12,13], which investigated the antimicrobial effects of numerous ligands and their ferric complexes, found that the ferric complex displayed higher antimicrobial activity against the selected bacteria and fungi than the functional ligand.

The ferric sillen core-linked polymer (FSCLP) is proposed as a potential solution to combat pathogens like *C. jejuni* in the gastrointestinal tract of animals. What makes an FSCLP distinctive is its structure and potential mechanism of action. Unlike organic acids, which may dissociate before reaching the lower gastrointestinal tract (GIT), an FSCLP potentially possess enhanced stability due to the strength of the complex formed. The primary objective of this study was to determine whether the FSCLP possessed inhibitory properties against the growth of *C. jejuni*. Additionally, real-time quantitative reverse transcription polymerase chain reaction (qRT-PCR) was used to examine the in vitro effect of sub-inhibitory concentrations of the FSCLP on gene expression in *C. jejuni*, in particular as regards those genes associated with virulence factors such as adhesion, invasion, iron metabolism, and toxin production, all of which are critical factors for infection success. The FSCLP was also assessed to determine whether it impacted *Campylobacter* host–cell interactions in vitro, utilising the intestinal porcine epithelial (IPEC-J2) cell line.

## 2. Materials and Methods

### 2.1. Strains and Culture Conditions

*C. jejuni* (DSMZ, Braunschweig, Germany, 24306) suspension was prepared from a liquid stock obtained from DSMZ. One hundred microliters of the stock was aseptically transferred to 20 mL of fresh sterile Bolton broth (BB) (Thermo Fisher Scientific, Illkirch-Graffenstade, France) supplemented with a Bolton broth selective antibiotic supplement (Thermo Fisher Scientific, Illkirch-Graffenstade, France). The culture was then incubated at 41.5 °C for 48 h under micro-aerobic conditions of 5% oxygen, 10% carbon dioxide, and 85% nitrogen (Concept 400, Baker Ruskinn, Bridgend, Wales) [14].

### 2.2. Production of a Ferric Sillen Core-Linked Polymer (FSCLP)

The FSCLP was produced essentially as outlined by [15], with subsequent modifications as outlined by [16].

### 2.3. Evaluation of the Minimum Inhibitory Concentration (MIC) Against C. jejuni

In this study, a modified version of the method developed by [17] was utilised. The fluorogenic indicator dye Alamar Blue^®^ (Thermo Fisher Scientific, Illkirch-Graffenstade, France) was used to determine the MIC values of the FSCLP against *C. jejuni.* A starter culture was generated as outlined above. The seed culture was then incubated at 41.5 °C for 24 ± 4 h. Bacterial cells were collected by centrifugation at 4000 rpm for 10 min at room temperature. The cell pellet was resuspended in BB to an OD 600 nm of 0.1 (2 × 10^8^ CFU mL^−1^). The new suspension (100 µL) was transferred to each well of a 96-well plate, containing 100 µL of the FSCLP (containing Fe at final concentrations of 0, 1.25, 2.5, 5, 10, 20, 25 ppm), and the positive control 100 µL of medium was added. The negative control well consisted of uninoculated medium. The plate was incubated for 48 h at 41.5 °C in a micro-aerobic environment. Following the incubation period, 20 µL Alamar Blue^®^ reagent was added to each well. Fluorescence was measured 4 h later at a ratio of 530 nm excitation/590 nm emission, using the BIO-TEK Synergy HT microtiter plate reader (Winooski, VT, USA). The average values of the three biological replicates were calculated, and the relative cell viability was calculated as the percentage of the untreated cells or the 0 ppm Fe supplement.

### 2.4. Reverse Transcription Real-Time Quantitative PCR (RT-qPCR)

Cultures of *C. jejuni* (20 mL, 1 × 10^8^ CFU mL^−1^) were grown in the presence of the FSCLP at a concentration of 1.25 ppm Fe, while the positive controls were grown in BB alone. Samples were taken over three different time points (4, 8, and 24 h) at 41.5 °C in a micro-aerobic environment; samples were then pelleted and washed twice with 1 mL PBS and snap-frozen in liquid nitrogen. RNA was extracted from pelleted cells using TRIzol^®^ Max™ Bacterial RNA Isolation Kit (Thermo Fisher Scientific, Illkirch-Graffenstade, France) according to the manufacturer’s instructions. RNA concentrations and purities were determined by diluting the RNA in sterile water and measuring the 260 to 280 nm absorbance ratios using a Nanodrop™ 2000/2000c Spectrophotometer (Thermo Fisher Scientific, Illkirch-Graffenstade, France). RNA integrity and quality was analysed with the Qubit RNA IQ Assay (Thermo Fisher Scientific, Illkirch-Graffenstade, France) according to the manufacturer’s instructions.

Reverse transcription of RNA (1 µg) was carried out using SuperScript™ III First-Strand Synthesis SuperMix according to the manufacturer’s instructions (Thermo Fisher Scientific, Illkirch-Graffenstade, France). The oligonucleotide primer sequences utilised in this study for the amplification of individual gene sequences are outlined in Table 1. The amplification specificity was tested using melt curve analysis. The amplified product was detected using SYBR Green reagent. Relative gene expression was determined using the comparative critical threshold (Ct) method on an Applied Biosystems 7500 Fast Real-Time PCR system (Foster City, CA, USA) using a method previously described by [18]. Each real-time PCR mixture (10 µL) contained 300 nM forward primer and 300 nM reverse primer, 2 µL of cDNA (10 ng), and 5 µL of 2× SYBR Green PCR Master Mix (Applied Biosystems Waltham, Massachusetts). The cycling parameters utilised were as follows: the holding stage which consists of a temperature of 50 °C for 2 min followed by 95 °C for 2 min. The next stage comprised 40 cycles of denaturation at 95 °C for 3 s and primer annealing/extension at 60 °C for 30 s. After amplification, the cycle threshold (C_T_) values of the target and endogenous control gene (*aspA*) were converted to raw quantities using the 2^−∆∆^Ct method to measure relative quantification [19]. The cDNA samples were processed individually, and the results from the 2^−∆∆^Ct calculation were averaged. The results were then plotted as a log_10_ transformation.

### 2.5. Cell Culture Conditions

IPEC-J2 intestinal porcine epithelial cells (IPEC-J2, DSMZ, Braunschweig, Germany) were maintained in Dulbecco′s Modified Eagle′s Medium (DMEM)—high glucose (Merck, Darmstadt, Germany)—with added 1% (*v*/*v*) L-glutamine (Thermo Fisher Scientific, France) and 10% (*v*/*v*) foetal bovine serum (FBS) (Thermo Fisher Scientific, France) and incubated at 37 °C in a humidified atmosphere containing 5% carbon dioxide (Forma™ Steri-Cycle™ CO_2_ Incubator, Thermo Fisher Scientific, France). Cells were passaged just prior to confluence every 3 to 4 days following removal with trypsin [20].

### 2.6. Infection Assay

The adhesion and invasion of *C. jejuni* to IPEC-J2 cells was investigated using a method outlined by [21] with the following modifications. IPEC-J2 cells were seeded under test conditions at a level of 2 × 10^5^ cells/well in 12 well cell culture plates and incubated overnight at 37 °C with 5% CO_2_. Following the incubation, the cell monolayer was washed three times with 1 mL of pre-warmed DMEM. The FSCLP was (1 mL) suspended in DMEM and was then added to the IPEC-J2 at a concentration of 1.25 ppm of Fe and incubated for 24 h at 37 °C in a 5% CO_2_ environment to prime the IPEC-J2 cells prior to infection. Following the priming stage, cells were washed three times with 1 mL of pre-warmed DMEM. *C. jejuni* culture was suspended in CO_2_-independent media supplemented with added 1% (*v*/*v*) L-glutamine, 10% (*v*/*v*) FBS, and FSCLP at a concentration of 1.25 ppm of Fe. *C. jejuni* was added to the IPEC-J2 cells at a concentration of 1 × 10^8^ CFU mL^−1^ and cells and bacteria were co-incubated in microaerobic conditions for 4 h at 37 °C. After infection, the cells were washed three times with 1 mL of pre-warmed CO_2_-independent media to remove non-adherent bacteria.

### 2.7. Invasion Assay

To determine the bacterial invasion, the IPEC-J2 cells were treated with 250 μg/mL^−1^ gentamicin (Merck, Darmstadt, Germany) in CO_2_-independent media (Merck, Darmstadt, Germany) supplemented with added 1% (*v*/*v*) L-glutamine and 10% (*v*/*v*) FBS for 2 h at room temperature to kill any remaining extracellular bacteria before triple washing with pre-warmed CO_2_-independent media. Subsequently, cells were lysed with 1 mL of 1% Triton X-100 in sterile 1X PBS for 5 min at room temperature. Following dilution in PBS, released intracellular bacteria were enumerated as described above. After microaerophilic incubation at 41.5 °C for 24 h, the colonies were counted to calculate the number of *C. jejuni* cells which had invaded the IPEC-J2 cells.

### 2.8. Adhesion Assay

To calculate the number of bacterial cells that had adhered to the IPEC-J2 cells surface, the washed monolayer was subsequently lysed with 1 mL of 1% Triton X-100 (Merck, Darmstadt, Germany) in sterile 1× PBS for 5 min at room temperature. The cell lysates were diluted using PBS and spread on CCDA plates to determine total bacteria (intra- and extracellular bacteria). The number of bacteria per sample was determined by counting CFU levels after 24 h incubation at 41.5 °C. To calculate the total adherent *C. jejuni* cells, the number of *C. jejuni* cells which invaded the IPEC-J2 cells (intracellular bacteria) was subtracted from the total intra- and extracellular bacteria.

### 2.9. Statistical Analysis

All data during this study were analysed using the Minitab statistical software package version 21.2 (Coventry, UK). One-way ANOVA with Dunnett’s test was used to determine whether there was a significant difference (*p* ≤ 0.05) between the untreated control sample and the treated sample in assessing the impact of FSCLP on growth, as well as its effect on adhesion to and invasion of IPEC-J2 cells. Real-time PCR experimental data were analysed using Student’s *t* test (*t* test) of the replicate 2^−∆∆^Ct values for each gene in the control group and treatment group; *p* < 0.05 was considered statistically different.

## 3. Results

### 3.1. Evaluation of Minimum Inhibitory Concentration (MIC) of FSCLP Against C. jejuni Using Alamar Blue^®^

**Figure 1:**
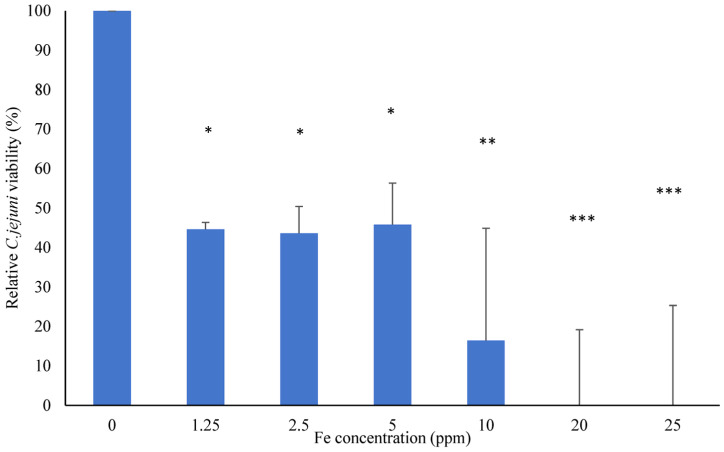
The inhibition of *C. jejuni* growth using an FSCLP. Cells were incubated for 48 h with the FSCLP at a range of Fe concentrations. Data are expressed as the mean with standard deviation (SD) of triplicate samples, with negative values adjusted to 0% to ensure biological accuracy. Statistically significant differences were determined to the corresponding positive control by one-way ANOVA followed by Dunnett’s test (denoted by * *p* < 0.05, ** *p* < 0.01, and *** *p* < 0.001).

The FSCLP resulted in significantly lower (*p* < 0.05) *Campylobacter* viability by approximately 45% at concentrations as low as 1.25 ppm of Fe, with the MIC calculated to be 20 ppm of Fe (Figure 1).

### 3.2. FSCLP’s Impact on the Expression Levels of C. jejuni Virulence Genes

The FSCLP concentration used for this experiment was determined based on the findings presented in Figure 1, which identified 1.25 ppm Fe as the lowest concentration within the sub-lethal range to optimise the quantity and quality of the RNA extraction from the *C. jejuni* cells. The presence of the FSCLP resulted in significantly greater expression levels of genes encoding the outer membrane ferric enterobactin receptor (*cfrA*) and the periplasmic iron-binding protein (*p19*) after 4 h. However, after 8 and 24 h, a reversal of this trend was observed, and all genes analysed were significantly downregulated; see Table 2.

### 3.3. The Impact of the FSCLP on the Ability of C. jejuni to Attach to or Invade Porcine Intestinal Cells (IPEC-J2)

**Figure 2:**
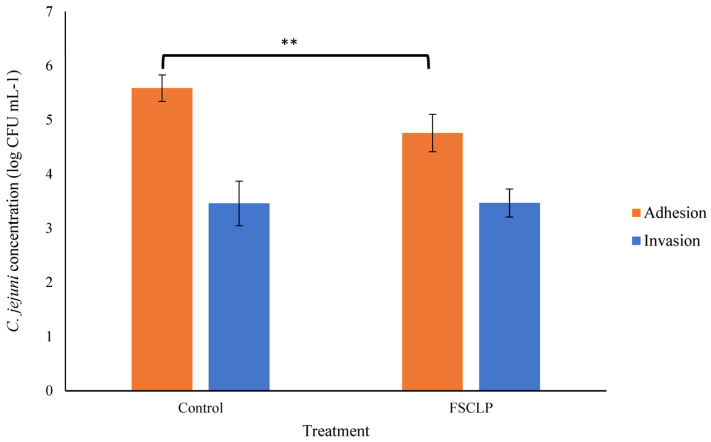
The influence of the FSCLP on *C. jejuni* adhesion to and the invasion of IPEC-J2 cell monolayers. Statistically significant differences were determined relative to the corresponding positive control (with no treatment) by one-way ANOVA followed by Dunnett’s test (denoted by ** *p* < 0.01). *n* = 3 for the adhesion and invasion assays.

The ability of *C. jejuni* to adhere to and invade biotic surfaces was investigated using IPEC-J2 cells treated with the FSCLP. The FSCLP was included at a sub-inhibitory concentration of 1.25 ppm of Fe in the culture medium throughout the infection assay; this compound resulted in significantly (*p* < 0.01) lower *C. jejuni* adhesion to IPEC-J2 cells (Figure 2). The number of *C. jejuni* cells that had adhered to IPEC-J2 cells was lowered from 5.59 log_10_ CFU mL^−1^ in the untreated IPEC-J2 cells to 4.76 log_10_ CFU mL^−1^ with the FSCLP treatment, representing a significant, almost six-fold lower attachment of *C. jejuni* cells. However, the FSCLP had no influence on *C. jejuni* invasion (Figure 2). The toxicity of the FSCLP to the IPEC-J2 cells was also assessed and no impact on cell viability was noted at 1.25 ppm of the compound; data are not presented here.

## 4. Discussion

The development of novel metallo-antimicrobials based on the capacity of active organic molecules to readily coordinate iron ions might be a viable way to ameliorate antimicrobial activity against *Campylobacter*. The present results demonstrate that bacterial growth was inhibited with the FSCLP at concentrations as low as 1.25 ppm of Fe. The resazurin reduction assay, using Alamar Blue, was used to confirm an expected lack of metabolic activity, showing that *C. jejuni* cells had died rather than entered a viable but nonculturable state. This method also provides a promising alternative to standard plating procedures due to its speed and its ability to be replicated in a microtiter plate, allowing for high throughput screening [17] also utilised this method to confirm the absence of metabolic activity in *C. fetus* cells.

Regarding the gene expression response of *C. jejuni* following the FSCLP treatment, changes were observed in gene expression at several time points spanning 24 h of growth. Genes involved in adhesion, the invasion into the host cell, toxin production, iron utilisation, and the response to environmental stress were analysed. Differences in gene expression with the treatment were transient (Table 2). This phenomenon was also observed in a study of *C. jejuni* by [22], where a substantial increase in temperature elicited similar changes in gene expression.

*C. jejuni* competes with other microbial iron sequestration and iron acquisition processes within the host to thrive. This is achieved through a number of iron absorption systems that work in tandem to utilise the various forms of iron present. These range from free ferrous iron to ferric forms bound by proteins such as transferrin or lactoferrin, heme iron, and a variety of small-molecular-weight chelators known as siderophores, which are generated by other bacteria within the gut microbiota [23]. Uncontrolled iron uptake can cause iron toxicity and oxidative stress, leading to an interruption in growth levels. Hence, the maintenance of iron homeostasis is critical. Throughout this investigation, the genes linked to ferric uptake were given particular attention, as the compound under examination is synthesised using a ferric source. In particular, attention was directed towards the gene linked with the outer membrane ferric enterobactin receptor, encoded by *cfrA*, the gene associated with an outer membrane receptor for transferrin or lactoferrin, *cfpbA*, and the *p19* gene, which encodes a periplasmic binding protein whose function is to transport iron. The results revealed there was an initial significant up-regulation in the expression of the ferric enterobactin transporter gene (*cfrA*) and *p19* (Table 2). Similar results were also seen in a study by [24], where under stress conditions, *cfrA* gene expression was similarly up-regulated along with other genes controlled by the ferric uptake regulator. The reason for the up-regulation of iron is purported to be a mechanism which maintains intracellular iron levels during stressful conditions [25]. However, at the 8 and 24 h time points, the expression of all genes linked to iron uptake or regulation in the present study were significantly down-regulated. This suppressed expression of iron uptake or regulation may be a method for *C. jejuni* to limit excessive iron intake and hence protect cells from oxidative stress caused by intracellular iron overload.

*Campylobacter* adhesion to the host intestinal epithelium is required for successful colonisation. *C. jejuni* has several distinct adhesins that, alone or together, can impact or mediate bacterial adherence to various cell structures and hosts. The most-studied adhesin is CadF (*Campylobacter* adhesin to fibronectin), which is encoded by the *cadF* gene. This adhesin binds to the extracellular matrix protein fibronectin, allowing interaction with integrin receptors, facilitating bacterial internalisation into host cells [26]. In this study, there was an initial non-significant up-regulation in the expression of *cadF* after 4 h; however, the following two time points (8 and 24 h) showed a significant down-regulation in expression. According to [27], under stress conditions, the *cadF* gene was up-regulated at 2, 4, and 6 h following starvation stress, and then down-regulated at 12, 24, and 48 h to the same expression level as seen at 0 h. Based on the findings of the current study, it can be hypothesised that stress may act as a signal for inducing *cadF*, which may play a significant role in *C. jejuni* pathogenicity and biofilm formation, and may act as an initial fight response; however, after the 4 h time point in this study, the down-regulation of this *C. jejuni* virulence factor demonstrated a reduced capacity to survive and colonise.

This study additionally looked at *ciaB* (*Campylobacter* invasive antigen B), a gene which is known to be involved in the translocation of *Campylobacter* into host cells for the purpose of host cell invasion and plays a significant role in caecal colonisation in chicken [28]. Treating *C. jejuni* with the FSCLP resulted in *ciaB* having an initial non-significant up-regulation in expression after 4 h, similar to *cadF*, but it was significantly down-regulated for the next two time points evaluated. Ref. [29] saw an up-regulation in *ciaB* expression when cells were grown in Fe-limited medium; therefore, the greater amount of Fe from the FSCLP treatment may be responsible for the down-regulation of the *ciaB* gene.

Cytolethal distending toxin (CDT), a genotoxin produced by *C. jejuni*, is composed of three subunits: CdtA, CdtB, and CdtC. CDT causes cell cycle arrest during the G2/M stage in eukaryotic cells. The binding and internalisation of *Campylobacter* into the host cell is mediated by CdtA and CdtC. Subsequently, CdtB enters the nucleus and demonstrates a DNase I-like activity, leading to DNA double-strand breaks [30]. The present study is primarily concerned with investigating the impact of FSCLP on the expression of the *cdtB* gene in *C. jejuni*, as *cdtB* is the enzymatically active component of the toxin [31]. Following 8 and 24 h of incubation, a significant reduction in the expression of the *cdtB* gene was observed in *C. jejuni* upon exposure to the FSCLP. These results suggest that the exposure of *C. jejuni* to the FSCLP has the potential to hamper its virulence [32].

The two colonisation-related genes, *racR* and *dnaJ*, have been identified as regulators of heat stress response in *C. jejuni*, and have been implicated in cell adhesion [30]. RacR represents a pivotal constituent of the RacRS two-component system, exhibiting responsiveness to changes in temperature. The significance of RacR is underscored by its prominent involvement in avian colonisation and growth at 42 °C, the physiological temperature of chickens, thereby emphasising its pivotal role in *C. jejuni* pathogenicity. Moreover, the dysregulation of the RacRS operon, under in vitro conditions, mimicking the infection situation of *C. jejuni*, independent of temperature fluctuations between 37 °C and 42 °C, suggests that RacR may possess multifaceted functions in *C. jejuni* physiology, colonisation, and potential contributions to pathogenesis [33]. Another important gene associated with *C. jejuni* thermotolerance is *dnaJ*. Investigations utilising a *C. jejuni dnaJ* mutant have revealed its inability to colonise newly hatched Leghorn chickens, demonstrating the critical role of heat shock proteins, including DnaJ, in facilitating successful in vivo infection establishment. This underscores the significance of *dnaJ* in mediating *C. jejuni*’s resilience to temperature-induced stress conditions [34]. Both *racR* and *dnaJ* play crucial roles in *C. jejuni*’s ability to adapt to temperature fluctuations and establish successful colonisation in avian hosts. The down-regulation in the expression of these two genes (Table 2) indicates that FSCLP may be capable of affecting *C. jejuni* colonisation.

Finally, the impact of the FSCLP on the ability of *C. jejuni* to adhere to and invade IPEC-J2 cells was assessed. The non-transformed IPEC-J2 cell line has been frequently utilised as a model for monogastrics and the human gut [35]. Adhesion to the intestinal epithelium is a prerequisite step for colonisation for many pathogenic bacteria, including *Campylobacter* [36]. The influence of the FSCLP on this process was examined and it was observed that the FSCLP significantly hampered the adherence of *C. jejuni* to the cell surface. However, lower *C. jejuni* adherence did not necessarily correlate with a corresponding lowering in cellular invasion. Similar results from earlier studies confirm this trend, which was observed in our investigation. For example, in swine intestinal epithelial cells (PSI cl.1 and CLAB), *Lactobacillus rhamnosus* LGG was demonstrated to dramatically reduce *C. jejuni* adherence, but this did not result in an equivalent reduction in bacterial invasion [36]. This may imply that different molecular or cellular pathways control adhesion and invasion, and that treatments aimed at adhesion may not necessarily have an impact on invasion. Such findings illustrate the complex nature of *C. jejuni*’s interaction with epithelial cells.

## 5. Conclusion

In conclusion the FSCLP demonstrated the ability to inhibit the growth of *C. jejuni* in vitro. Additionally, lowering the expression of key genes associated with virulence and colonisation was also observed, which further supports the noted phenotypic impacts on growth. The results also showed the positive impact of FSCLP on reducing the adhesion of *C. jejuni* to IPEC-J2 cells. However, while these findings are promising, it is imperative to acknowledge the need for further research, specifically in the form of animal studies, to corroborate the efficacy of this metallo-antimicrobial in ameliorating diseases associated with *C. jejuni* in monogastric animals.

## Figures and Tables

**Table 1:** The primer sequences used to amplify the target and internal control genes from treated and untreated *C. jejuni* cDNA by RT-qPCR.

Gene	Direction	Accession No.	Primer Sequence (5′–3′)
*aspA*	F	KC405084	TGAAAGGTTCAAAATGGGAACAAGA
	R		AACCTCATCAGAGATTTCCAATTCC
*cfrA*	F	CP001876	CCAGGCGTTGATTTATATGC
	R		CCCATCAATCAAAACCAAAG
*p19*	F	CP001876	CAATCCAAATGGTTTTCCAG
	R		ATTGCACCTGTGTCGGTATT
*ciaB*	F	CP054847	AAAAGCTTGGCAAGAAGCTG
	R		ATGCCACCGCATGAGTATAA
*cfbpA*	F	CP001876	TAGCTTCGCCTGCAAATACT
	R		GGCTAAAGGTGTGCTTGAAA
*cadF*	F	KJ875965	TTCTATGGTTTAGCAGGTGGAGG
	R		CCGCGCCATAATGTCCAAA
*cdtB*	F	KX495592	CGCAGCCACAGAAAGCAAAT
	R		AGCTCCTACATCAACGCGAG
*racR*	F	KJ477104	TGTGGGGCTTCAAATCGGT
	R		CAACTCTTTTTGTGCGACGA
*dnaJ*	F	KY792734	AGTGTCGAGCTTAATATCCC
	R		GGCGATGATCTTAACATACA

F = forward primer; R = reverse primer. The *aspA* gene was used as an endogenous control for the expression quantification.

**Table 2:** Analysis of *C. jejuni* gene expression when grown in presence of FSCLP.

	log_10_ Fold Change			
Gene	4 h	8 h	24 h	Gene Function
cfrA	**0.03**	**−0.10**	**−0.38**	Ferric enterobactin receptor
p19	**0.19**	**−0.18**	**−0.18**	Ferric acquisition
ciaB	0.23	**−0.16**	**−0.23**	Invasion
cfbpA	0.06	**−0.26**	**−0.42**	Ferric acquisition
cadF	0.07	**−0.17**	**−0.17**	Adhesion
cdtB	0.01	**−0.30**	**−0.46**	Toxin production
racR	0.20	**−0.20**	**−0.26**	Response to heat shock
dnaJ	0.17	**−0.22**	**−0.23**	Response to hyperosmotic and heat shock

Statistical analysis was undertaken using Student’s *t* test of 2^−∆∆^Ct values for each gene in control group compared to treatment groups, and significant differences (*p* < 0.05) are indicated in bold (*n* = 3).

## Data Availability

The data supporting the findings of this study are proprietary and cannot be publicly shared. For inquiries regarding access to the data, please contact the corresponding author, Seán Christanseen (schristanseen@alttech.com).

## References

[1] Kuhn K.G., Nielsen E.M., Mølbak K., Ethelberg S. (2018). Epidemiology of Campylobacteriosis in Denmark 2000–2015. Zoonoses Public Health.

[2] Kirk M.D., Pires S.M., Black R.E., Caipo M., Crump J.A., Devleesschauwer B., Döpfer D., Fazil A., Fischer-Walker C.L., Hald T. (2015). World Health Organization Estimates of the Global and Regional Disease Burden of 22 Foodborne Bacterial, Protozoal, and Viral Diseases, 2010: A Data Synthesis. PLoS Med..

[3] Liu F., Lee S.A., Xue J., Riordan S.M., Zhang L. (2022). Global Epidemiology of Campylobacteriosis and the Impact of COVID-19. Front. Cell. Infect. Microbiol..

[4] Awada R., Ghssein G., Roz A.E., Farhat M., Nehme N., Hassan H.F. (2023). Prevalence of *Campylobacter* Spp. in Broilers in North Lebanon. Vet. World.

[5] Ibrahim J.N., Eghnatios E., El Roz A., Fardoun T., Ghssein G. (2019). Prevalence, Antimicrobial Resistance and Risk Factors for Campylobacteriosis in Lebanon. J. Infect. Dev. Ctries..

[6] Kaakoush N.O., Castaño-Rodríguez N., Mitchell H.M., Man S.M. (2015). Global Epidemiology of *Campylobacter* Infection. Clin. Microbiol. Rev..

[7] Klancnik A., Guzej B., Jamnik P., Vucković D., Abram M., Mozina S.S. (2009). Stress Response and Pathogenic Potential of *Campylobacter jejuni* Cells Exposed to Starvation. Res. Microbiol..

[8] Kreling V., Falcone F.H., Kehrenberg C., Hensel A. (2020). *Campylobacter* Sp.: Pathogenicity Factors and Prevention Methods—New Molecular Targets for Innovative Antivirulence Drugs?. Appl. Microbiol. Biotechnol..

[9] Ben-Othman S., Jõudu I., Bhat R. (2020). Bioactives from Agri-Food Wastes: Present Insights and Future Challenges. Molecules.

[10] Gómez-García M., Sol C., de Nova P.J.G., Puyalto M., Mesas L., Puente H., Mencía-Ares Ó., Miranda R., Argüello H., Rubio P. (2019). Antimicrobial Activity of a Selection of Organic Acids, Their Salts and Essential Oils against Swine Enteropathogenic Bacteria. Porc. Health Manag..

[11] Dittoe D.K., Ricke S.C., Kiess A.S. (2018). Organic Acids and Potential for Modifying the Avian Gastrointestinal Tract and Reducing Pathogens and Disease. Front. Vet. Sci..

[12] Soliman S.M., Al-Rasheed H.H., Albering J.H., El-Faham A. (2020). Fe(III) Complexes Based on Mono- and Bis-Pyrazolyl-s-Triazine Ligands: Synthesis, Molecular Structure, Hirshfeld, and Antimicrobial Evaluations. Molecules.

[13] Chandio A.A., Ali Memon A., Memon S., Memon F.N., Panhwar Q.K., Durmaz F., Nizamani S.M., Brohi N.A. (2019). Synthesis and Antimicrobial Assessment of Fe^3+^ Inclusion Complex of P-Tert-Butylcalix[4]Arene Diamide Derivative. J. Chem..

[14] Davis L., DiRita V. (2008). Growth and Laboratory Maintenance of *Campylobacter jejuni*. Curr. Protoc. Microbiol..

[15] Zhang C., Miao M., Cao X., An Z. (2012). One-Pot RAFT Synthesis of Core Cross-Linked Star Polymers of polyPEGMA in Water by Sequential Homogeneous and Heterogeneous Polymerizations. Polym. Chem..

[16] Christanseen S. (2023). Targeted *Campylobacter* Control: The Development and Analysis of Novel Feed Additives on *Campylobacter* Growth. Ph.D. Thesis.

[17] Graham L.L., Feero S.E. (2019). The *Campylobacter* Fetus S Layer Provides Resistance to Photoactivated Zinc Oxide Nanoparticles. Can. J. Microbiol..

[18] Schmittgen T.D., Livak K.J. (2008). Analyzing Real-Time PCR Data by the Comparative CT Method. Nat. Protoc..

[19] Livak K.J., Schmittgen T.D. (2001). Analysis of Relative Gene Expression Data Using Real-Time Quantitative PCR and the 2^−∆∆C_T_^ Method. Methods.

[20] Schierack P., Nordhoff M., Pollmann M., Weyrauch K.D., Amasheh S., Lodemann U., Jores J., Tachu B., Kleta S., Blikslager A. (2006). Characterization of a Porcine Intestinal Epithelial Cell Line for in Vitro Studies of Microbial Pathogenesis in Swine. Histochem. Cell Biol..

[21] Boehm M., Krause-Gruszczynska M., Rohde M., Tegtmeyer N., Takahashi S., Oyarzabal O.A., Backert S. (2011). Major Host Factors Involved in Epithelial Cell Invasion of *Campylobacter jejuni*: Role of Fibronectin, Integrin Beta1, FAK, Tiam-1, and DOCK180 in Activating Rho GTPase Rac1. Front. Cell. Infect. Microbiol..

[22] Stintzi A. (2003). Gene Expression Profile of *Campylobacter jejuni* in Response to Growth Temperature Variation. J. Bacteriol..

[23] Stahl M., Butcher J., Stintzi A. (2012). Nutrient Acquisition and Metabolism by *Campylobacter jejuni*. Front. Cell. Infect. Microbiol..

[24] Askoura M., Youns M., Halim Hegazy W.A. (2020). Investigating the Influence of Iron on *Campylobacter jejuni* Transcriptome in Response to Acid Stress. Microb. Pathog..

[25] Birk T., Wik M.T., Lametsch R., Knøchel S. (2012). Acid Stress Response and Protein Induction in *Campylobacter jejuni* Isolates with Different Acid Tolerance. BMC Microbiol..

[26] Sierra-Arguello Y.M., Perdoncini G., Rodrigues L.B., Ruschel dos Santos L., Apellanis Borges K., Quedi Furian T., Pippi Salle C.T., de Souza Moraes H.L., Pereira Gomes M.J., Pinheiro do Nascimento V. (2021). Identification of Pathogenic Genes in *Campylobacter jejuni* Isolated from Broiler Carcasses and Broiler Slaughterhouses. Sci. Rep..

[27] Ma Y., Hanning I., Slavik M. (2009). Stress-Induced Adaptive Tolerance Response and Virulence Gene Expression in *Campylobacter jejuni*. J. Food Saf..

[28] Konkel M.E., Kim B.J., Rivera-Amill V., Garvis S.G. (1999). Bacterial Secreted Proteins Are Required for the Internalization of *Campylobacter jejuni* into Cultured Mammalian Cells. Mol. Microbiol..

[29] Holmes K., Mulholland F., Pearson B.M., Pin C., McNicholl-Kennedy J., Ketley J.M., Wells J.M.Y. (2005). *Campylobacter jejuni* Gene Expression in Response to Iron Limitation and the Role of Fur. Microbiology.

[30] Lai C.-K., Chen Y.-A., Lin C.-J., Lin H.-J., Kao M.-C., Huang M.-Z., Lin Y.-H., Chiang-Ni C., Chen C.-J., Lo U.-G. (2016). Molecular Mechanisms and Potential Clinical Applications of *Campylobacter jejuni* Cytolethal Distending Toxin. Front. Cell. Infect. Microbiol..

[31] Johansson C., Nilsson A., Kaden R., Rautelin H. (2019). Differences in Virulence Gene Expression between Human Blood and Stool *Campylobacter* Coli Clade 1 ST828CC Isolates. Gut Pathog..

[32] Jinadasa R.N., Bloom S.E., Weiss R.S., Duhamel G.E. (2011). Cytolethal Distending Toxin: A Conserved Bacterial Genotoxin That Blocks Cell Cycle Progression, Leading to Apoptosis of a Broad Range of Mammalian Cell Lineages. Microbiology.

[33] Apel D., Ellermeier J., Pryjma M., DiRita V.J., Gaynor E.C. (2012). Characterization of *Campylobacter jejuni* RacRS Reveals Roles in the Heat Shock Response, Motility, and Maintenance of Cell Length Homogeneity. J. Bacteriol..

[34] Konkel M.E., Kim B.J., Klena J.D., Young C.R., Ziprin R. (1998). Characterization of the Thermal Stress Response of *Campylobacter jejuni*. Infect. Immun..

[35] Xu X., Yan G., Chang J., Wang P., Yin Q., Liu C., Liu S., Zhu Q., Lu F. (2020). Astilbin Ameliorates Deoxynivalenol-Induced Oxidative Stress and Apoptosis in Intestinal Porcine Epithelial Cells (IPEC-J2). J. Appl. Toxicol. JAT.

[36] Šikić Pogačar M., Langerholc T., Mičetić-Turk D., Možina S.S., Klančnik A. (2020). Effect of *Lactobacillus* Spp. on Adhesion, Invasion, and Translocation of *Campylobacter jejuni* in Chicken and Pig Small-Intestinal Epithelial Cell Lines. BMC Vet. Res..

